# Bioinformatic analysis and experimental validation identified DNA methylation–Related biomarkers and immune-cell infiltration of atherosclerosis

**DOI:** 10.3389/fgene.2022.989459

**Published:** 2022-09-08

**Authors:** Congjian Xu, Di Sun, Changmin Wei, Hao Chang

**Affiliations:** ^1^ Department of Cardiology, Shengli Oilfield Central Hospital, Dongying, Shandong, China; ^2^ Hanyu Biomed Center Beijing, Beijing, China

**Keywords:** DNA methylation, diagnosis, MS-PCR, immune-cell infiltration, atherosclerosis

## Abstract

**Background:** DNA methylation is an important form of epigenetic regulation and is closely related to atherosclerosis (AS). The purpose of this study was to identify DNA methylation–related biomarkers and explore the immune-infiltrate characteristics of AS based on methylation data.

**Methods:** DNA methylation data of 15 atherosclerotic and paired healthy tissues were obtained from Gene Expression Omnibus database. Differential methylation positions (DMPs) and differential methylation regions (DMRs) were screened by the ChAMP R package. The methylation levels of DMPs located on CpG islands of gene promoter regions were averaged. The limma R package was used to screen differentially methylated genes in the CpG islands of the promoter regions. The diagnostic values of the methylation levels were evaluated using the pROC R package. The EpiDISH algorithm was applied to quantify the infiltration levels of seven types of immune cells. Subsequently, three pairs of clinical specimens of coronary atherosclerosis with Stary’s pathological stage III were collected, and the methylation levels were detected by the methylation-specific PCR (MS-PCR) assay. Western blot was performed to detect the protein expression levels of monocyte markers.

**Results:** A total of 110, 695 DMPs, and 918 DMRs were screened in the whole genome. Also, six genes with significant methylation differences in the CpG islands of the promoter regions were identified, including 49 DMPs. In total, three genes (GRIK2, HOXA2, and HOXA3) had delta beta greater than 0.2. The infiltration level of monocytes was significantly upregulated in AS tissues. MS-PCR assay confirmed the methylation status of the aforementioned three genes in AS samples. The Western blot results showed that the expression levels of the monocyte marker CD14 and M1-type macrophage marker CD86 were significantly increased in AS while M2-type macrophage marker protein CD206 was significantly decreased.

**Conclusion:** This study identified potential DNA methylation–related biomarkers and revealed the role of monocytes in early AS.

## Introduction

Atherosclerosis (AS) is a chronic disease with extensive studies (Libby et al., 2019). It is no doubt that AS is continuously developing worldwide, and there are emerging challenges. For example, AS has been found in the younger population and females. In addition, new risk factors have been found besides diets, such as physical inactivity, microbiome, and epigenetics (Xu et al., 2018; Libby, 2021). All of which guide the new strategies for the prevention, diagnosis, and treatment of AS.

Chronic AS may lead to acute cardiovascular events (Ahmadi et al., 2019). The timely diagnosis of AS is critical. Until now, the monitoring of AS plaque development and rupture has been still most effective, since it causes most cardiovascular diseases in clinical practice (Rader and Daugherty, 2008; Mushenkova et al., 2020). However, instead of plaque monitoring, the early diagnosis of AS has been more important in preventing the disease and subsequent outcomes (Herrington et al., 2016). Inflammation and immunity-related biomarkers have been reported to link with traditional and emerging risk factors, including pro-inflammatory cytokines, inflammatory signaling pathways factors, bioactive lipids, and adhesion molecules (Zhu et al., 2018; Libby, 2021). Recently, more evidence suggests that AS is an epigenetic disease and its development involves several epigenetic processes, including DNA methylation, histone modification, and non-coding RNAs (Xu et al., 2018).

As a chronic disease, the pathogenesis of AS can be classified into three processes (Hai and Zuo, 2016; Tabaei and Tabaee, 2019). First, the activation of endothelial cells, infiltration of monocytes and formation of foam cells. Second, the stimulation of smooth muscle cells (SMCs), followed by differentiation, migration, and phenotypic switching of immune cells, thus forming the plaque. Third, the rupture of AS plaque and the occurrence of thrombosis. Several mechanisms and regulatory pathways participate in the long and complex processes during AS development, which may involve various epigenetic regulations (Khyzha et al., 2017; Tang et al., 2021).

DNA methylation is the epigenetic event that covalently transfers a methyl group to the cytosine, mainly on the CpG dinucleotide site and CpG islands, regulating the expression of specific target genes at the transcriptional level (Moore L. D. et al., 2013). Genomic DNA methylations have been proved to play a crucial part in the early progression of AS (Dong et al., 2002; Zhang et al., 2021). For example, DNA methylation can regulate or partially regulate different AS-related genes, such as ERα/β, MMP9/2/7, EC-SOD, INF-γ, and eNOS. These genes were targets of inflammatory response and reaction, macrophages, apoptosis, cell proliferation, and differentiation (Hai and Zuo, 2016).

CpG islands are GC-rich and primarily located at the 5′ regulatory regions of all housekeeping genes (Deaton and Bird, 2011). Aberrant DNA methylation in CpG islands would upregulate AS-susceptible genes and downregulate AS-protective genes, contributing to the AS progression (Dong et al., 2002). Varied DNA methylation aberrations have been reported during the development of AS. The exploration of this information may be significant for better understanding AS pathogenesis. Furthermore, some epigenetic drugs with therapeutic potential may be screened (Xu et al., 2018; Zhang et al., 2021).

In this study, bioinformatic analysis has been performed on the DNA methylation data of AS patients. Differential methylation positions (DMPs) and differential methylation regions (DMRs) were systematically screened. Then differentially methylated genes in the CpG islands of the promoter regions were identified. Furthermore, the infiltration levels of different immune cells were quantified for analyzing the immune status of AS. Finally, the DNA methylation of target genes and variations of immune cells have been confirmed experimentally in samples of AS patients.

## Materials and methods

### Methylome dataset

**FIGURE 1:**
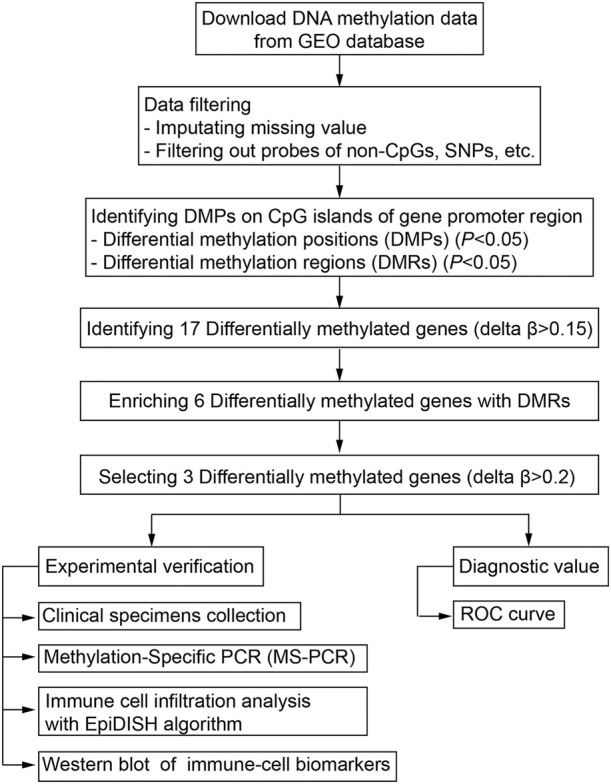
Workflow of the bioinformatic analysis for screening aberrant DNA methylation sites.

The DNA methylation dataset GSE46394 including 15 atherosclerotic and paired healthy tissues was obtained from the Gene Expression Omnibus (GEO) database (https://ncbi.nlm.nih.gov/gds). The methylation levels were evaluated based on the Illumina Infinium Human Methylation 450 Beadchip platform and quantified as a β-value. The mean values of methylated (M) and non-methylated (U) signal intensity for each tissue and CpG sites (CpGs) were calculated using the formula [*β* = M/(M + U)]. The workflow for screening aberrant DNA methylation has been presented (Figure 1).

### DNA methylation differential analyses

After the imputation of missing values with the function of impute.knn in the impute package of R, ChAMP (Tian et al., 2017) was used for the follow-up analyses. First, probes that belong to non-CpGs, including SNPs (Zhou et al., 2017), align to multiple locations, and located on X are Y chromosomes were filtered out with the champ.filter function. Then the filtered data were normalized with the BMIQ method for types I and II probe correction by the champ.norm function. Differential methylation positions (DMPs) with a BH adjusted *p*-value below 0.05 were screened using the champ.DMP function. Differential methylation regions (DMRs) were identified using the Bumphunter method with a *p*-value below 0.05 by using the champ.DMR function. The org.Hs.eg.db R package was then used to annotate the genes corresponding to these DMRs with UCSC.hg19 as the reference genome file. The function enrichments were performed with Metascape (www.metascape.org).

### Methylation analysis of CpG islands in gene promoter regions

The *β*-value was defined as the value of the methylation expression profiles, with range between 0 (no methylation) and 1 (complete methylation). The average β-value of DMPs located on CpG islands of the gene promoter region was calculated. The limma R package (Ritchie et al., 2015) was used to screen differentially methylated genes in the CpG islands of the promoter regions. The diagnostic values of the methylation levels were evaluated with receiver operating characteristic (ROC) established using the pROC package in R (Robin et al., 2011).

### Clinical specimens and ethical statement

A total of three pairs of clinical specimens of coronary atherosclerosis with Stary’s pathological stage III were collected from the Central Hospital of Shengli Oil Field (Yutani et al., 1999). All patients signed the informed consent form, and the study was approved by the Scientific Research Ethics Committee of the Central Hospital of Shengli Oil Field.

### Methylation-specific PCR

MS-PCR based on bisulfite conversion was conducted. Genomic DNA from three pairs of clinical specimens of coronary atherosclerosis with Stary’s pathological stage III was isolated using the DNA extraction kit (Beyotime, China), and the premium bisulfite kit (Diagenode, Belgium) was applied for sodium bisulfite treatment of the genomic DNA, according to the manufacturer’s protocol. The MS-PCR primers used in this study were designed using the Methyl Primer Express v1.0 and listed in Table 1.

**TABLE 1:** Sequences of MSP primers.

Gene	Primer sequence	
GRIK2	Methylation	
	Forward (5′–3′)	Reverse (5′–3′)
	TCG​CGT​TTT​TTT​TTT​TTT​C	ACT​AAT​AAT​CCT​CAC​ACG​CG
	No methylation	
	Forward (5′–3′)	Reverse (5′–3′)
	TTA​TTG​TGT​TTT​TTT​TTT​TTT​T	CTA​ACT​AAT​AAT​CCT​CAC​ACA​CA
HOXA2	Methylation	
	Forward (5′–3′)	Reverse (5′–3′)
	TAT​TTT​TTT​GGT​TGG​TCG​TC	AAA​CGA​CTC​TCG​AAA​CTT​CC
	No methylation	
	Forward (5′–3′)	Reverse (5′–3′)
	GGT​ATT​TTT​TTG​GTT​GGT​TGT​T	AAA​AAC​AAC​TCT​CAA​AAC​TTC​C
HOXA3	Methylation	
	Forward (5′–3′)	Reverse (5′–3′)
	GGA​TTA​GAC​GTT​GTT​TCG​C	CCC​GAA​AAT​AAA​CGC​TAA​T
	No methylation	
	Forward (5′–3′)	Reverse (5′–3′)
	GGA​TTA​GAT​GTT​GTT​TTG​T	CCC​AAA​AAT​AAA​CAC​TAA​T

### Immune-cell infiltration analysis

Based on the methylation β-value of CpGs, the EpiDISH R package (Zheng et al., 2019) was applied to quantify the infiltration levels of seven types of immune cells, including B cells, NK cells, CD4^+^ T cells, CD8^+^ T cells, monocytes, neutrophils, and eosinophils. The difference analysis was performed using the Wilcoxon test.

### Western blot

The expression levels of proteins were determined by Western blotting. The tissues were homogenized with RIPA lysis buffer (25 mM Tris-HCl, pH 7.6, 150 mM NaCl, 1% NP-40, 1% deoxycholic acid, and 0.1% SDS) for extracting proteins, and the proteins were separated using SDS-PAGE and transferred to PVDF membrane (Millipore, Germany). Then after blocking with 5% skim milk, the membrane was incubated with primary antibodies for overnight at 4°C. The applied primary antibodies were as follows: CD14 (1:1,000, ab106285, Abcam), CD86 (1:1,000, ab220188, Abcam), and CD206 (1:1,000, K006619P, Solarbio). The HRP-labeled secondary antibody was incubated for 2 h at room temperature. The ECL Western blotting detection system (Tanon, China) and ImageJ software were applied to visualize and analyze the results.

### Statistics

Methylation data were analyzed in R 4.1.2 software, and WB data were analyzed using Prism 9.0 software. Student’s *t*-test was used for two groups. *p* < 0.05 was indicated to be statistically significant.

## Results

### Differential methylation positions

**FIGURE 2:**
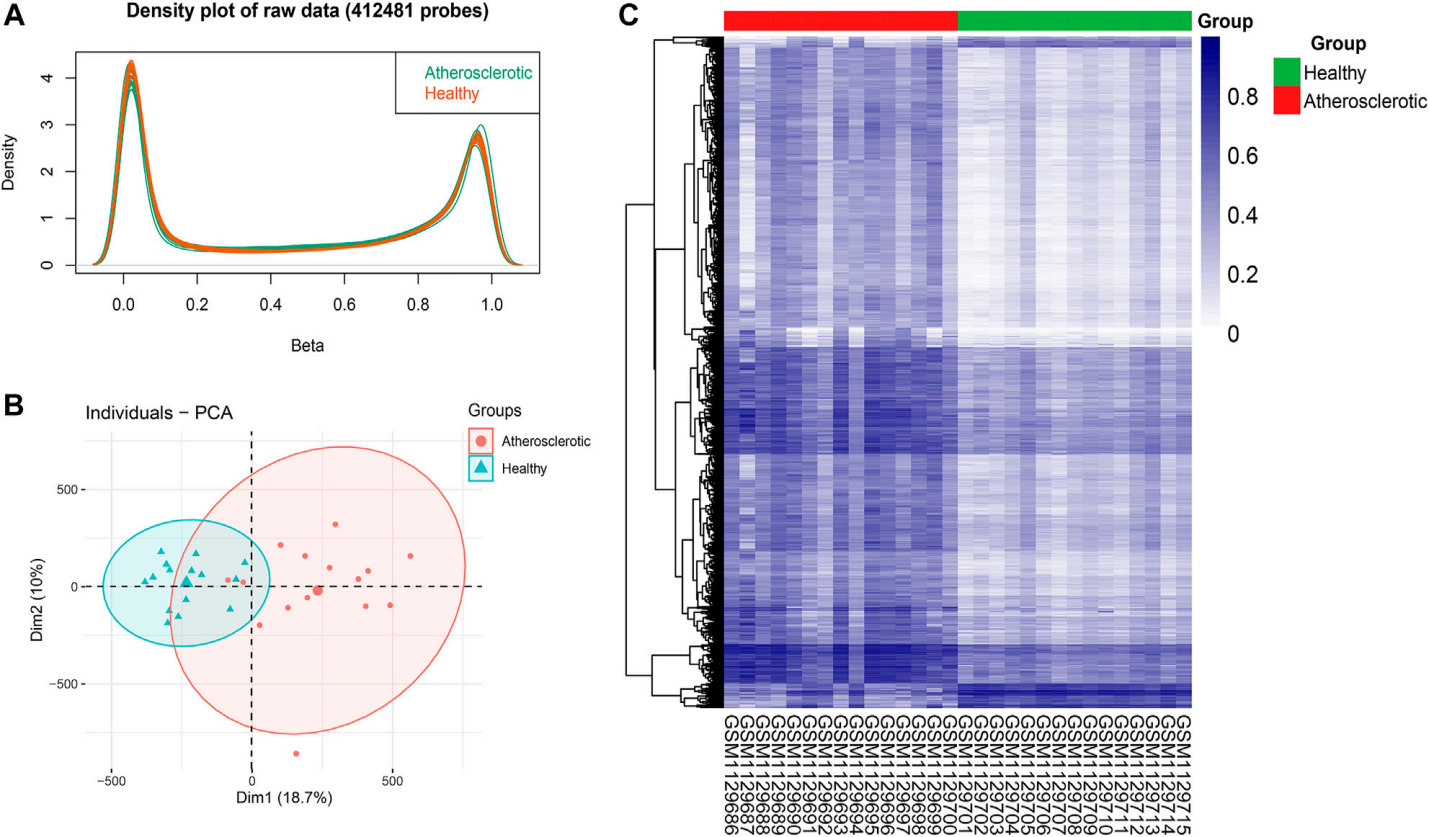
Identification of differential methylation positions (DMPs) in atherosclerosis. **(A)** Density plot of 412,481 probes’ raw data. **(B)** Principal component analysis (PCA) of the normalized data in the atherosclerotic and healthy groups. **(C)** Heat map of DMPs with β-value variance in the top 1,000.

After the imputation of missing values, probes that belong to non-CpGs (*n* = 3,156), including SNPs (*n* = 59,901), align to multiple locations (*n* = 11), and located on X and Y chromosomes (*n* = 10,028) were filtered out using the champ.filter function. The raw data of 412,481 probes were density plotted (Figure 2A). Then the data were normalized using the BMIQ method for types I and II probe correction (Teschendorff et al., 2013). The principal component analysis (PCA) of the normalized data in the atherosclerotic and healthy groups was performed (Figure 2B). A total of 110695 significant DMPs with a BH adjusted *p*-value below 0.05 were identified using the champ.DMP function. Heat maps of DMPs with β-value variance in the top 1,000 were drawn using the pheatmap R package (Figure 2C). Most DMPs were hypermethylated.

### Differential methylation regions

**FIGURE 3:**
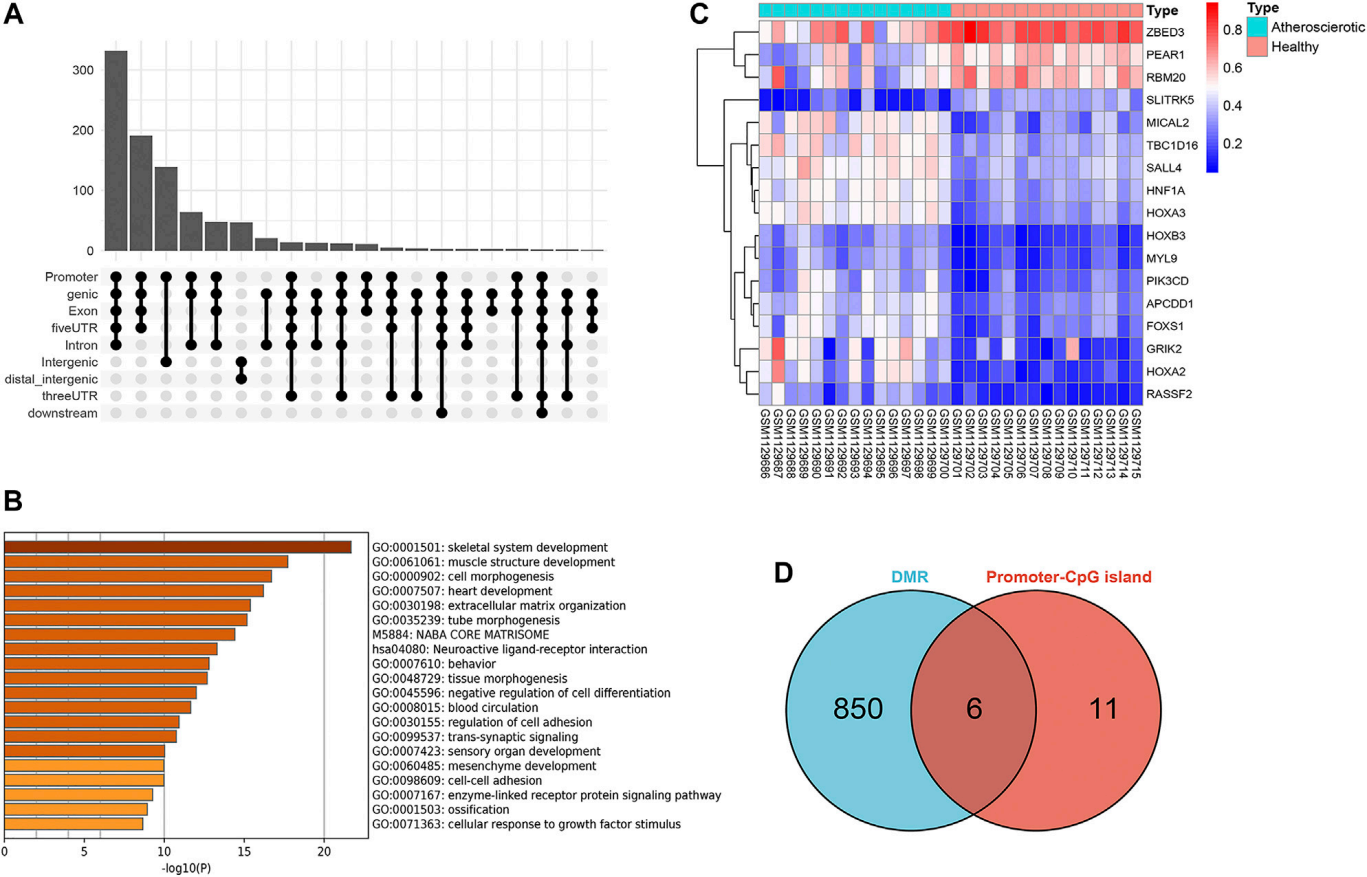
Screening of gene promoter regions enriched with differential methylation regions (DMRs). **(A)** Upset diagram of DMRs’ distribution. **(B)** Functional enrichment analysis with Metascape. **(C)** Heat map of the 17 genes identified with delta β over 0.15. **(D)** Venn diagram identified six genes which were enriched with DMRs.

A total of 918 differential methylation regions (DMRs) were identified using the Bumphunter method with a *p*-value below 0.05 by using the champ.DMR function (Supplementary File S1). We use UpSetR package to plot the upset diagram of these DMRs distribution regions. Most DMRs are distributed in promoter, genic, exon, and 5′ UTR regions (Figure 3A). These DMRs were annotated to 854 genes, and a functional enrichment analysis revealed that the functions of these genes are mainly concentrated in skeletal system development, muscle structure development, cell morphogenesis, heart development, extracellular cell matrix organization, tube morphogenesis, and so on (Figure 3B).

### Methylation analysis of CpG islands in gene promoter regions

Since the existing low-throughput methylation detection techniques, such as MS-PCR and bisulfite sequencing PCR (BS-PCR), and other classical methods are designed based on the CpG islands of gene promoter regions (Li, 2007); we further estimated the methylation levels of gene promoter regions to select markers with a practical application value. The average β-value of DMPs located on CpG islands of the gene promoter region was calculated. In total, 17 genes were identified with delta β over 0.15, and the heat map was shown (Figure 3C). The details of differential methylation data are shown in Table 2. In addition, six of these genes (HOXA2, GRIK2, HOXA3, TBC1D16, MYL9, and HNF1A) were enriched with DMRs (Figure 3D). Next, we selected three genes (HOXA2, GRIK2, and HOXA3) with delta β over 0.20 to conduct experimental verification.

**TABLE 2:** Characteristics of genes with significantly difference methylation levels in promoters.

Gene	AS mean	Healthy mean	Delta β	CpGs hits	adj *p* value
HOXA2	0.429	0.156	0.273	7	2.43E-05
GRIK2	0.435	0.216	0.219	10	2.49E-03
MICAL2	0.495	0.278	0.217	2	3.24E-05
HOXA3	0.498	0.295	0.203	19	7.86E-07
RASSF2	0.307	0.124	0.182	1	8.51E-05
FOXS1	0.423	0.241	0.182	2	4.51E-05
TBC1D16	0.519	0.348	0.171	3	8.87E-06
HOXB3	0.305	0.147	0.159	7	6.86E-06
MYL9	0.323	0.165	0.158	3	2.43E-05
HNF1A	0.480	0.323	0.157	7	8.29E-06
PIK3CD	0.375	0.220	0.155	2	5.45E-04
SALL4	0.513	0.361	0.153	1	6.63E-06
APCDD1	0.422	0.272	0.150	1	2.61E-05
PEAR1	0.414	0.581	−0.167	1	4.24E-04
RBM20	0.450	0.623	−0.173	2	2.07E-03
SLITRK5	0.156	0.347	−0.191	3	4.51E-05
ZBED3	0.595	0.795	−0.200	1	4.11E-04

### Exploration of the diagnostic biomarkers in methylation levels with methylation-specific PCR

**FIGURE 4:**
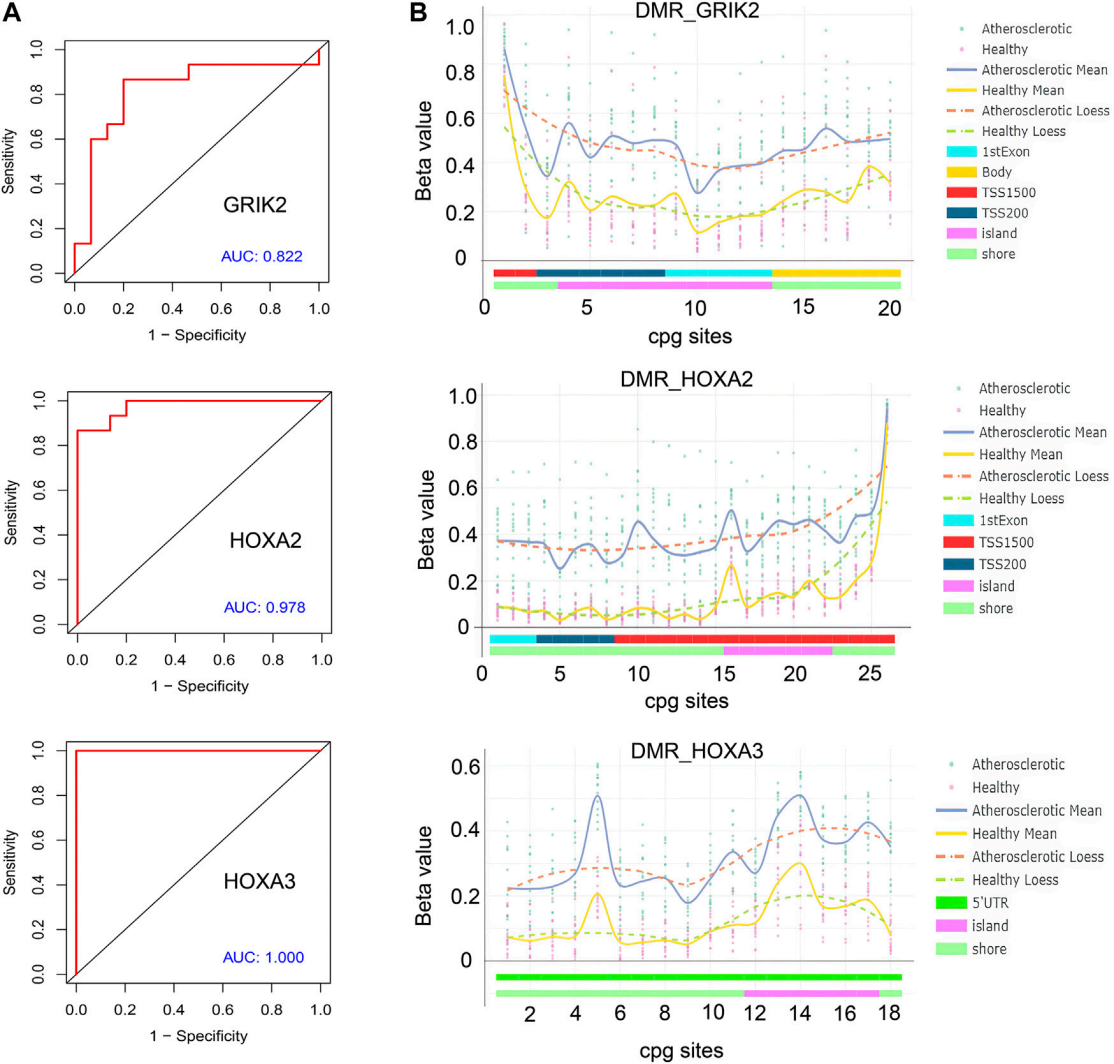
Bioinformatic analysis of the identified genes’ diagnostic values. **(A)** Receiver operating characteristic (ROC) curves of GRIK2, HOXA2, and HOXA3. **(B)** β-value and distribution characteristics of CpG sites within the DMRs located in GRIK2, HOXA2, and HOXA3.

**FIGURE 5:**
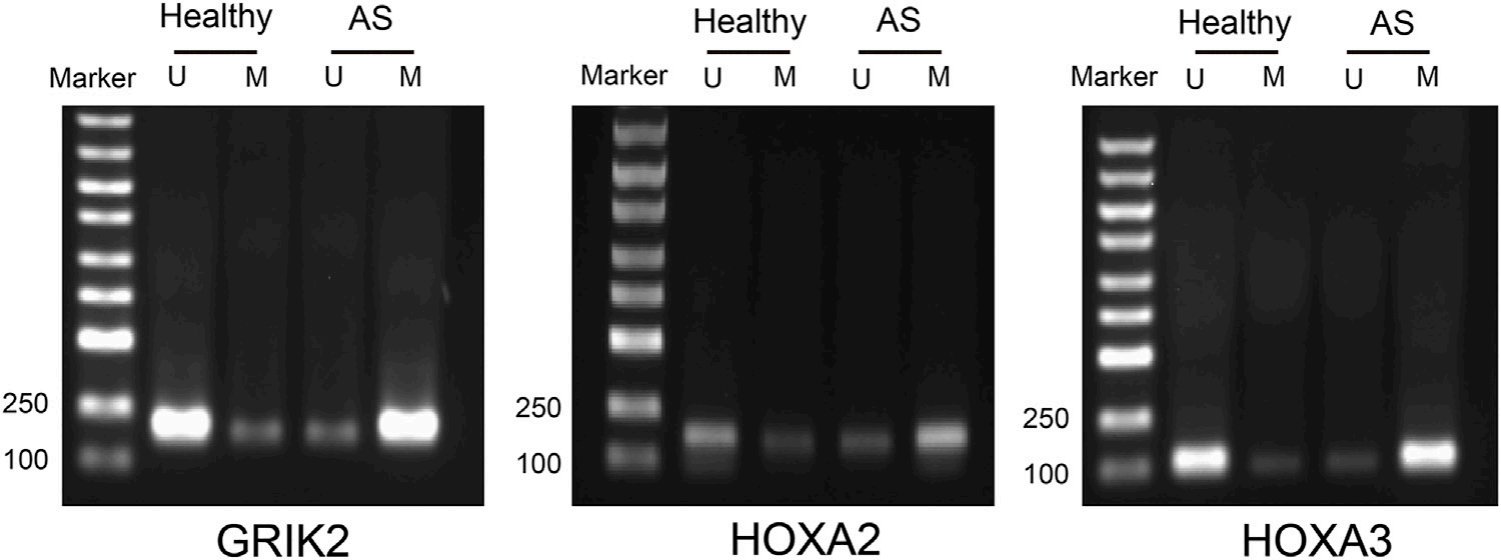
Methylation-specific PCR detection of the methylation statuses in the CpG islands of the promoter regions in atherosclerosis and healthy samples. U, un-methylated; M, methylated.

The diagnostic values of the identified three genes were evaluated with the receiver operating characteristic (ROC) curves. The area under curve (AUC) values were 0.822 for GRIK2, 0.978 for HOXA2, and 1.0 for HOXA3 separately (Figure 4A). The β-value and distribution characteristics of CpG sites within the DMRs located in these genes were plotted (Figure 4B). The characteristics of CpG sites located on the islands of gene promoter regions are shown in Table 3. The MS-PCR results indicated that the promoters’ methylation statuses of all the three genes were almost un-methylated in the healthy group while partially methylated in the atherosclerotic group (Figure 5). These data confirmed the differences in the promoter regions and their potential diagnostic values.

**TABLE 3:** Characteristics of CpG sites located on the islands of gene promoter regions.

Gene	CpG sites	AS	Healthy	Delta β	adj *p* value	Feat cgi	UCSC CpG islands name
GRIK2	cg18193094	0.275	0.117	0.158	5.31E-04	1stExon island	chr6:101846766-101847135
	cg10591607	0.368	0.155	0.212	1.55E-03	1stExon island	chr6:101846766-101847135
	cg22541254	0.420	0.206	0.214	3.48E-03	TSS200 island	chr6:101846766-101847135
	cg24301620	0.473	0.273	0.199	5.16E-03	1stExon island	chr6:101846766-101847135
	cg13080565	0.478	0.230	0.248	5.38E-03	TSS200 island	chr6:101846766-101847135
	cg26316946	0.387	0.181	0.206	8.24E-03	1stExon island	chr6:101846766-101847135
	cg06247406	0.507	0.262	0.245	9.88E-03	TSS200 island	chr6:101846766-101847135
	cg05942459	0.491	0.227	0.264	1.12E-02	TSS200 island	chr6:101846766-101847135
	cg21635870	0.396	0.188	0.208	1.24E-02	1stExon island	chr6:101846766-101847135
	cg24753760	0.561	0.322	0.239	1.27E-02	TSS200 island	chr6:101846766-101847135
HOXA2	cg06166490	0.458	0.149	0.309	6.94E-07	TSS1500 island	chr7:27143181-27143479
	cg19432993	0.391	0.126	0.266	6.99E-07	TSS1500 island	chr7:27143181-27143479
	cg01217984	0.504	0.267	0.236	2.05E-06	TSS1500 island	chr7:27143181-27143479
	cg00445443	0.417	0.131	0.286	3.78E-06	TSS1500 island	chr7:27143181-27143479
	cg04027736	0.463	0.201	0.261	4.15E-06	TSS1500 island	chr7:27143181-27143479
	cg10319053	0.444	0.131	0.313	5.60E-06	TSS1500 island	chr7:27143181-27143479
	cg02225599	0.327	0.089	0.239	2.19E-05	TSS1500 island	chr7:27143181-27143479
HOXA3	cg09591524	0.652	0.384	0.268	2.87E-08	5′UTR island	chr7:27150030-27150418
	cg02439266	0.612	0.344	0.268	6.02E-08	5′UTR island	chr7:27150030-27150418
	cg26297005	0.562	0.246	0.316	7.07E-08	5′UTR island	chr7:27162087-27162426
	cg19999161	0.427	0.187	0.240	9.75E-08	5′UTR island	chr7:27154999-27155426
	cg18430152	0.713	0.349	0.365	9.75E-08	5′UTR island	chr7:27162087-27162426
	cg04778178	0.713	0.363	0.350	1.18E-07	5′UTR island	chr7:27162087-27162426
	cg04351734	0.373	0.168	0.205	1.04E-06	5′UTR island	chr7:27154999-27155426
	cg16748008	0.270	0.118	0.153	1.72E-06	5′UTR island	chr7:27154999-27155426
	cg22798849	0.366	0.169	0.197	1.84E-06	5′UTR island	chr7:27154999-27155426
	cg16406967	0.449	0.241	0.208	2.78E-06	5′UTR island	chr7:27154999-27155426
	cg18680977	0.510	0.301	0.209	9.42E-06	5′UTR island	chr7:27154999-27155426
	cg07522913	0.351	0.220	0.132	2.72E-05	5′UTR island	chr7:27150030-27150418
	cg01301319	0.451	0.308	0.143	7.76E-05	5′UTR island	chr7:27153187-27153647
	cg13172549	0.446	0.252	0.193	8.64E-05	5′UTR island	chr7:27153187-27153647
	cg22962123	0.493	0.335	0.158	1.54E-04	5′UTR island	chr7:27153187-27153647
	cg09144964	0.398	0.286	0.112	5.72E-04	5′UTR island	chr7:27150030-27150418
	cg05851442	0.446	0.347	0.099	1.39E-03	5′UTR island	chr7:27153187-27153647
	cg24360871	0.733	0.579	0.154	1.44E-03	5′UTR island	chr7:27163819-27164098
	cg03536885	0.503	0.411	0.092	8.60E-03	5′UTR island	chr7:27163819-27164098

### The infiltration of monocytes and M1-type macrophages were significantly increased in atherosclerotic tissues at the pre-atheroma stage

**FIGURE 6:**
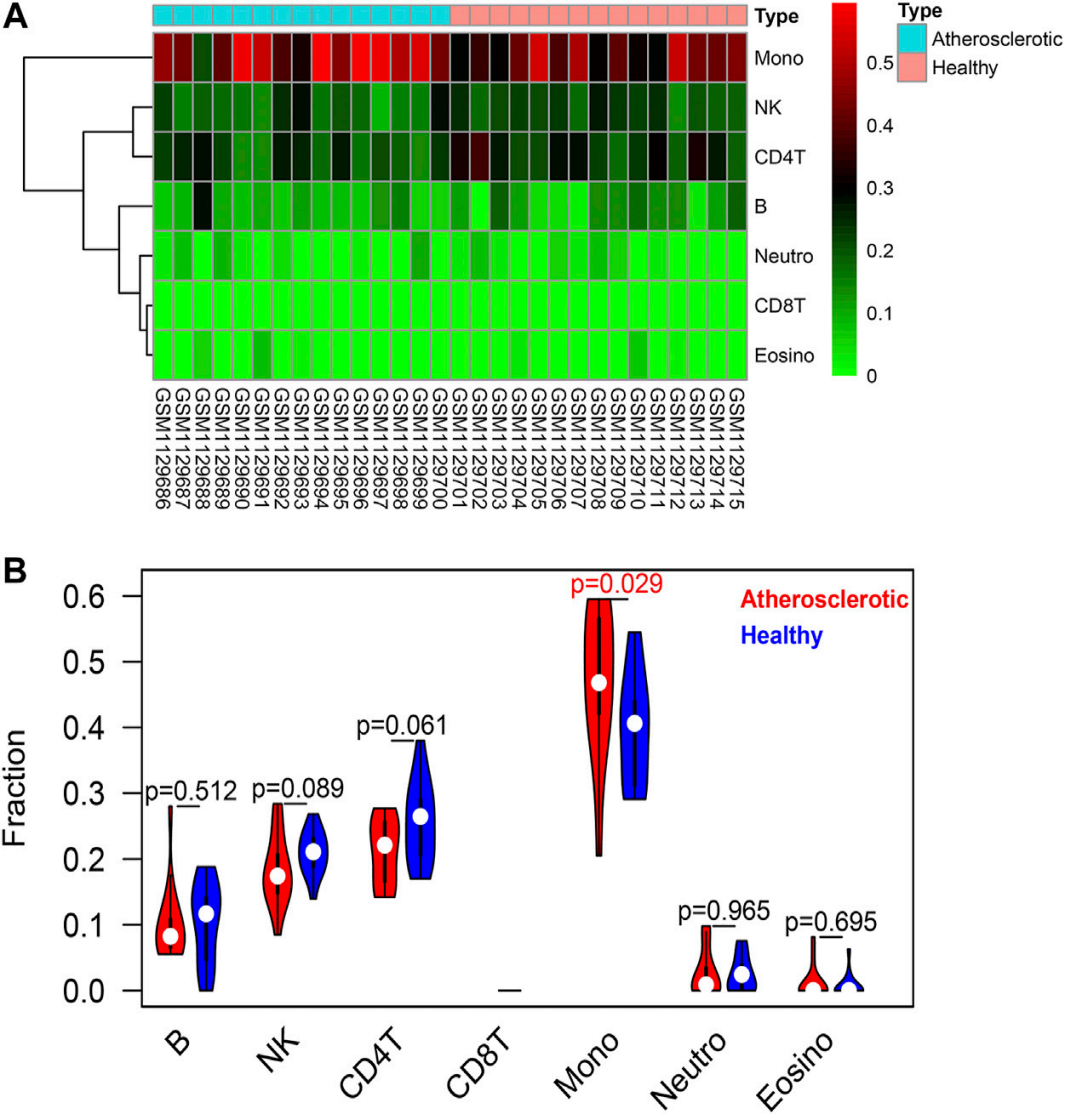
Immune-cell infiltration analysis based on the EpiDISH algorithm. **(A)** Heat map of the infiltration levels of seven types of immune cells (B cells, NK cells, CD4^+^ T cells, CD8^+^ T cells, monocytes, neutrophils, and eosinophils). **(B)** Violin plot and difference analysis between the atherosclerotic and healthy groups.

**FIGURE 7:**
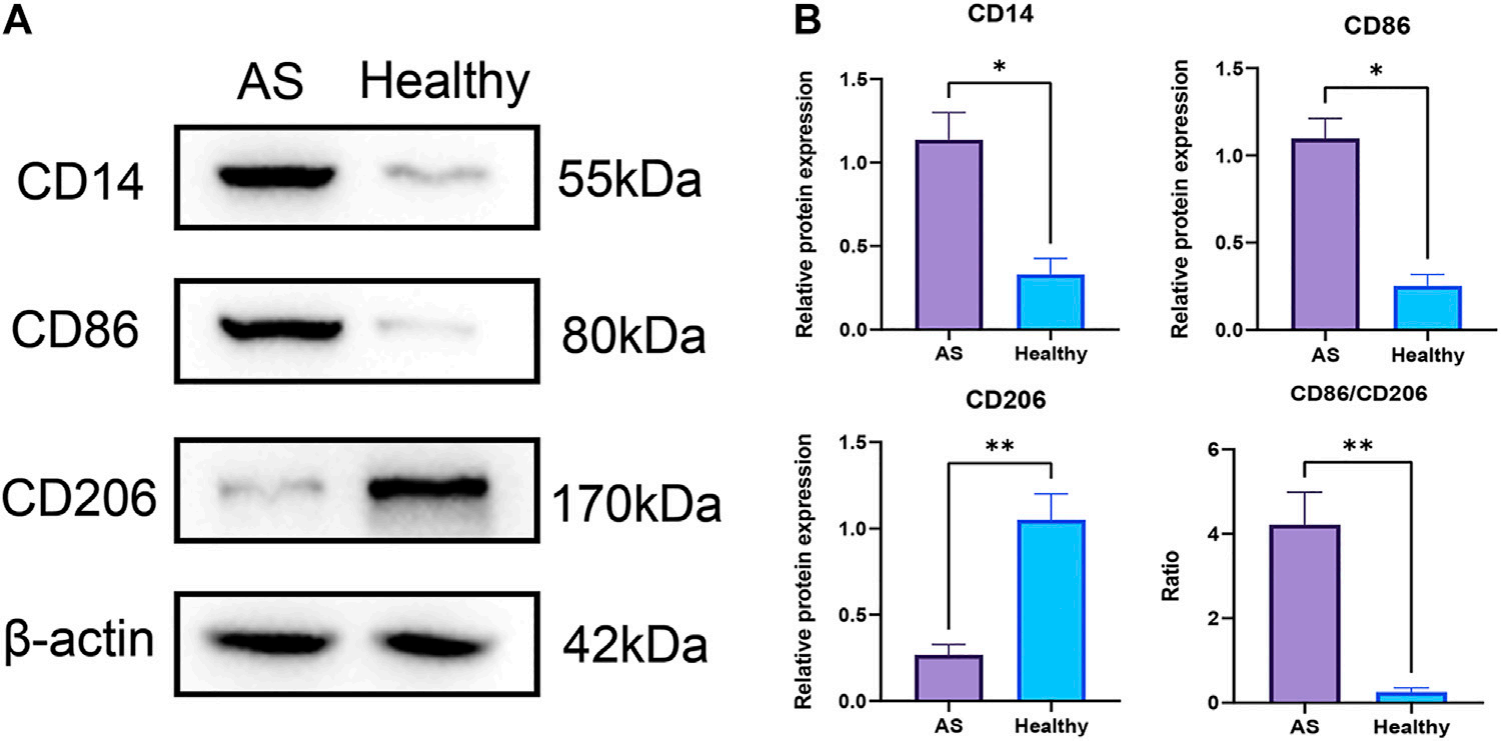
Infiltrations of monocytes and M1-type macrophages were significantly increased in atherosclerotic tissues at pre-atheroma stage. **(A)** Western blots of the monocyte marker CD14, M1-type macrophage marker CD86, and M2-type macrophage marker CD206. **(B)** Differential analyses of the relative protein expression levels. **: *p* < 0.01; *: *p* < 0.05.

The EpiDISH algorithm was used to assess immune-cell infiltration (Supplementary File S2). The heat map and violin plots are shown in Figures 6A,B separately. The results of immune-cell infiltration analysis showed that the infiltration degree of monocytes was significantly upregulated in AS tissues, whereas the infiltration level of NK cells and CD4T cells showed a downward trend, but there were no statistical differences (Figure 6B). It has been reported that macrophage polarization plays a key role in the progression of atherosclerosis, so we tested the expression of marker proteins (CD14, CD86, and CD206) by Western blot. The expression levels of monocyte marker CD14 and M1-type macrophage marker CD86 were significantly increased in AS while that of M2-type macrophage marker protein CD206 was significantly decreased (Figures 7A,B). The CD86/CD206 ratios were also significantly increased in AS (Figure 7B).

## Discussion

In this study, based on the DNA methylation data of AS patients downloaded from the database, a bioinformatic analysis has been performed for demonstrating the aberrant DNA methylations. A total of three differentially methylated genes (GRIK2, HOXA2, and HOXA3) in the CpG islands of the promoter regions have been screened. These genes can be applied as biomarkers for the early diagnosis of AS. The MS-PCR data proved that all the three genes were almost un-methylated in the healthy group while partially methylated in AS group. The immune-cell infiltration in AS patients was also investigated. The infiltration level of monocytes was significantly upregulated in AS tissues. The Western blot data indicated similar results; the expression levels of monocyte marker CD14 and M1-type macrophage marker CD86 were significantly increased while that of M2-type macrophage marker CD206 was significantly decreased, with significantly increased CD86/CD206 ratios. Both the diagnosis biomarkers and potential immune cell–relevant mechanisms were theoretically and experimentally revealed.

Recent studies have found that DNA methylations were related to the whole development process of AS (Borghini et al., 2013; Zaina, 2014). The potential roles of DNA methylation in AS have been reported to link with oxidative stress (Kalea et al., 2018), inflammation, SMCs, and homocysteine (Hcy) (Ma et al., 2017; Tabaei and Tabaee, 2019). Numerous studies have shown that oxidative stress affected DNA methylation during AS. At the presence of oxidative stress such as H_2_O_2_ and reactive oxygen species, DNA methyltransferase1 (DNMT1) may be relocalized from non-GC-rich to GC-rich areas. The uprising methylation level in CpG island may promote AS development (O’Hagan et al., 2011). Inflammation has been found in all stages of AS; thus, inflammatory molecules have been identified as markers of the AS progression (Zhu et al., 2018; Bäck et al., 2019). DNMT may regulate the expression of inflammatory factors by varying their DNA methylation, thus accelerating the progression of AS (Zhu et al., 2018). A bioinformatic study reported an imprinted gene PLA2G7, which encoded lipoprotein-associated phospholipase A2 (Lp-PLA2). The hypomethylation of PLA2G7 increased expression upon inflammation in AS, indicating the effects of DNA methylation modification on atheroprogression and destabilization *via* inflammatory processes (Li et al., 2021). SMCs migrate from the media to the intima and form the AS plaques. The DNA methylation of SMCs regulated their differentiation, migration, and phenotypic switching, thus influencing plaque formation (Tabaei and Tabaee, 2019). Furthermore, Hcy has been considered an independent risk factor for AS. Hcy can promote global DNA hypomethylation, which was reported to participate in endothelial dysfunction and SMC proliferation induced by DNA methylations (Ehrlich, 2019). However, another study performed on AS patients reported that the upregulated level of Hcy was positively correlated with more hypermethylation of CpG islands in the ER-α gene promoter region, as well as the severity of AS lesion. It has been further verified with *in vitro* experiments (Huang et al., 2009).

Since the DNA methylation and CpG islands methylation/demethylation matters in AS progress, several studies have tried to explore more potential DNA methylation sites and regions. There are generally three DNA methylation approaches for finding DNA methylation aberrations: candidate gene, global methylation, and epigenome-wide association studies (Fernández-Sanlés et al., 2017). However, there is still challenging to find critical DNA methylation aberrations effectively. The approach based on candidate gene seems to be low in throughput. For improving the throughput, the genome-wide DNA methylation aberrations have been obtained based on the methylation microarray chip. More than 400,000 methylated CpGs could be obtained with a typical Illumina Human Methylation 450 BeadChip. Even after setting criteria involving absolute β-value, there were still 1,458 differently methylated CpGs covering 971 genes were extracted (Chen et al., 2020). The high-throughput bioinformatic analysis seems to be more significant for pathway and mechanism analysis. In addition, in most of the recent studies involving global methylation, the DNA methylation has been explored based on the promoter regions of target genes while there are still abundant but unrevealed CpGs located on the non-promoter region. Thus, some researchers have tried to conduct synthetic analysis to improve the efficiency of screening significant candidate genes and corresponding DNA methylation. For example, in a study on DNA methylation and coronary heart disease including AS, a total of 51 individual articles were comprehensively analyzed (Fernández-Sanlés et al., 2017). Consistent results reported in at least two articles were identified. In addition, the probability of finding the same gene or CpG in two studies was calculated. With this strategy, some candidate genes were revealed, including hypermethylation in ESRα, ABCG1 and FOXP3, and hypomethylation in IL-6. These target genes were associated with several diseases and functions, including inflammatory, metabolic, and cardiovascular diseases (Zhu et al., 2018). In our study, the methylation levels of gene promoter regions were innovatively introduced for selecting markers with practical application value. The average β-value of DMPs located on CpG islands of the gene promoter region was calculated. A total of 17 genes identified with delta β over 0.15 and 6 were enriched. In total, three genes (HOXA2, GRIK2, and HOXA3) with delta β over 0.20 were further selected to conduct experimental verification. Since existing strategies for screening target genes and corresponding DNA methylation aberration have been still limited, more studies can be performed for providing new and effective theoretical or experimental tools.

Some studies have been performed to investigate the three genes (HOXA2, GRIK2, and HOXA3) identified as biomarker genes for AS. HOXA2 and HOXA3 were both homeobox genes, which were associated with the regulation of normal differentiation and development of cells. The homeobox genes have been reported to link with various cancers. Especially, hypermethylated CpG islands for HOXA gene promoters were associated with pathways in cancers. In colorectal cancer, the percentage of methylation of three HOXA genes (HOXA5, HOXA2, and HOXA6) were up to 67.62%, 58.36%, and 31.32%, respectively. The results demonstrated that colorectal cancer tissues and cells had a stronger methylation status around these three HOXA gene promoter regions, compared with adjacent controls. The epigenetic silencing of these three HOXA genes may be an important event in the progression of colorectal cancer (Li et al., 2019). The low-methylation epigenotype of HOXA2 and HOXA9 in squamous cell carcinoma was associated with idiopathic pulmonary fibrosis and poorer prognosis (Hata et al., 2020). In another study on prostate cancer, HOXA2, HOXA9, and HOXA10 were identified as critical genes, which were both abnormally expressed and associated with clinical outcomes of patients with prostate cancer (Song et al., 2022). In addition to cancers, the CpG methylation of HOXA2 was related to severe fibrosis and the progression in hepatitis B-related chronic liver disease (Zeybel et al., 2016). An early Russia study has also reported that HOXA2 exhibited most pronounced difference in the methylation level for it’s CpG sites. HOXA2 was found to be hypomethylated in the carotid atherosclerotic plaques compared to its methylation patterns in normal control veins (Nazarenko et al., 2013). Until now, few studies have reported the association between GRIK2 and atherosclerosis. GRIK2 gene encoded proteins belong to the kainate family of glutamate receptors. GRIK2 was involved in various normal neurophysiologic processes. The aberrant expression of GRIK2 has been reported to be involved in regions, functional genes, biological function, and pathways that mediate depression disorder. GRIK2 also showed abnormal methylation pattern specific to astrocytic dysfunction associated with depressive psychopathology (Nagy et al., 2015; Wang W. et al., 2021). It is interesting to explore the potential association of AS with the nervous system.

Immune infiltration is closely related to the progression and prognosis of AS (Wang L. et al., 2021; Tan et al., 2021). Several studies have tried to analyze the immune-cell infiltration profiles at different AS progresses. A review has suggested the importance of metabolic and functional reprogramming in monocytes and macrophages for AS progression. Furthermore, macrophages and monocyte contributed to pro- or anti-inflammatory mechanisms (Groh et al., 2018; Kim et al., 2020). Monocytes seemed to matter in early AS (Moroni et al., 2019). Blood monocytes expressed receptors for vascular endothelial growth factors for endothelial cells. After activation, monocytes were associated with inflammation by producing inflammatory molecules. Notably, monocytes consisted of distinct subsets with varied cell surface markers and functional characteristics, which may be relevant to angiogenic processes in AS (Jaipersad et al., 2014). The macrophages with four subtypes (M2a, M2b, M2c, and M2d) have different impacts on AS (Moore K. J. et al., 2013). M1 and M2 are strongly related to vascular calcification. Both the M1 and M2 phenotypes are found in the early and advanced lesions of AS. M1 macrophages predominated in unstable plaques, as AS developed, the number of M2 macrophages decreased (Bisgaard et al., 2016). However, the proportion of M2 macrophages in stable plaques was relatively higher and more M2 macrophages were required in plaque regression (Rahman et al., 2017; Yang et al., 2020). Our results have shown similarities with these conclusions, in AS tissues at the pre-atheroma stage, the expression levels of monocyte marker CD14 and M1-type macrophage marker CD86 were significantly increased, indicating higher immune filtration of monocyte and M1-type macrophage, while that of M2-type macrophage marker protein CD206 was significantly decreased, indicating a reduced ratio of M2-type macrophage. These results suggested the early formation of plaque.

Some studies have tried to explore the relationship between DNA methylation and immune infiltrate, which may assist in understanding the immune-related mechanism of certain diseases. In one study on hepatocellular carcinoma, a comprehensive analysis has been performed to explore the cell division cycle-associated family genes (CDCAs) methylation and immune infiltrates. The biological enrichment analysis of CDCAs demonstrated that they were significantly associated with the immune function regulation of infiltrating immune cells. Also, the methylation analysis of CDCAs indicated an association with the tumor immunogenicity, i.e., low-methylation of CDCA1, CDCA2, and CDCA8 dramatically reduced the immune infiltrate levels of T cells and cytotoxic lymphocytes. In addition, CDCA1-6 and CDCA8 with low-methylation levels significantly deteriorated the overall survival of HCC patients. It concluded that the methylation levels of CDCAs were related to the prognostic value and infiltrating immune differences, which could be a convincing biomarker for predicting the response of immunotherapy (Wang et al., 2020). In another study on hepatocellular carcinoma, LOXL3 was first reported to link with immune infiltrates. A statistical analysis has been applied to explore the relationship between the LOXL3 expression and the infiltration of multiple immune cells (Wang N. et al., 2021; Triki et al., 2022). The association between target gene and immune-cell infiltration can be further verified experimentally, with quantitative multiplex immunohistochemistry or immunohistochemistry staining (Wang Y. et al., 2021; Gatti et al., 2021).

There are still limitations in this study. One limitation is the sample resource. For the early diagnosis and prevention of AS, the expression profile of biomarkers during early AS should be focused on. Thus, the samples should be collected from the population without AS but developed into AS in the future, such as those with familial history of AS (Wright et al., 2021). However, most of the existing data have been derived from AS patients. Considering the complex progress during AS development, bias is inevitable. An early and lasting monitoring plan can be designed and conducted for obtaining more valuable data from people in the pre-AS stages. Another limitation is that the potential relationship between methylation of target genes and immune infiltration can be further explored. The statistical analysis should be preliminarily performed, and then, the relationship can be experimentally verified with immunohistochemistry. It may assist in understanding the roles of target genes in immuno-microenvironment during disease progression.

## Conclusion

Both the biomarkers and potential immune cell–relevant mechanisms were demonstrated with both bioinformatic analysis and experimental results. The bioinformatic analysis revealed three differentially methylated genes (GRIK2, HOXA2, and HOXA3) in the CpG islands of the promoter regions between healthy and AS groups. These genes can be applied as biomarkers for the early diagnosis of AS. For the AS tissues at the pre-atheroma stage, the expression levels of monocyte marker CD14 and M1-type macrophage marker CD86 were significantly increased while that of M2-type macrophage marker CD206 was significantly decreased. The specific immune-cell filtration conditions may also assist in understanding the progression of AS and finding the treatment targeting specific immune cells.

## Data Availability

The original contributions presented in the study are included in the article/Supplementary Material; further inquiries can be directed to the corresponding authors.
